# Urbach Energy: Film Processing and Thickness Effects

**DOI:** 10.1021/acs.langmuir.5c06770

**Published:** 2026-04-11

**Authors:** Marleane Maria Felix de Azevedo, Endel Ezequiel Carvalho Costa, Guilherme Severino Mendes de Araújo, Kayla Manuelli Pimentel de Carvalho, Cleânio da Luz Lima, Ángel Alberto Hidalgo, Maria Leticia Vega

**Affiliations:** † Departamento de Física − CCN, 67823 Universidade Federal do Piauí, Campus Universitário Ministro Petrônio Portella, Bairro Ininga, Teresina, Piauí CEP: 64049-550, Brazil; ‡ Instituto de Física “Gleb Wataghin” − IFGW, Universidade Estadual de Campinas (UNICAMP), Campinas, São Paulo CEP: 13083-970, Brazil; § Instituto Federal de Educação, Ciência e Tecnologia do Piauí (IFPI), Campus São Raimundo Nonato, BR 020, Bairro Primavera, São Raimundo Nonato, Piauí CEP: 64770-000, Brazil

## Abstract

Understanding how structural order, aggregation, and vibrational coupling influence the optical and electronic properties of conjugated polymers is essential for optimizing their performance in optoelectronic devices. Here, we compare partially ordered MEH-PPV films prepared by the Langmuir–Blodgett (LB) technique with structurally disordered films obtained by drop-casting, as well as with the polymer in solution. UV–vis absorption, steady-state photoluminescence, polarized spectroscopy, Raman scattering, and charge-transport measurements were employed. In solution, variations in the optical gap and Urbach tail with concentration reveal early aggregation effects, manifested in the absorption tail prior to significant perturbation of the conjugated backbone. LB films exhibit partial orientational order, confirmed by absorption dichroism and polarized emission, whereas drop-cast films show a similar optical response in perpendicular directions. Despite this orientational order, LB films show similar Urbach energies parallel and perpendicular to the dipping direction, indicating that the optical defect distribution is not direction-dependent, although a clear thickness dependence reveals the role of interface effects. Vibronic analysis (Huang–Rhys factor and effective conjugation length) demonstrates that LB deposition preferentially aligns polymer backbones with longer conjugation lengths and modifies the dominant vibrational modes coupled to emission, in agreement with Raman spectroscopy. Electrical current density vs electric field measurements further distinguish the two morphologies: partially ordered LB devices exhibit smoother and more stable transitions between ohmic and space-charge-limited current (SCLC), whereas disordered drop-cast devices reach the TFLC regime at lower fields, indicating a lower trap density distributed over a broader energy range. The comparison between optical (Urbach) and electrical (TFLC) analyses confirms that shallow optical traps and deep electrical traps probe distinct disorder populations.

## Graphical abstract

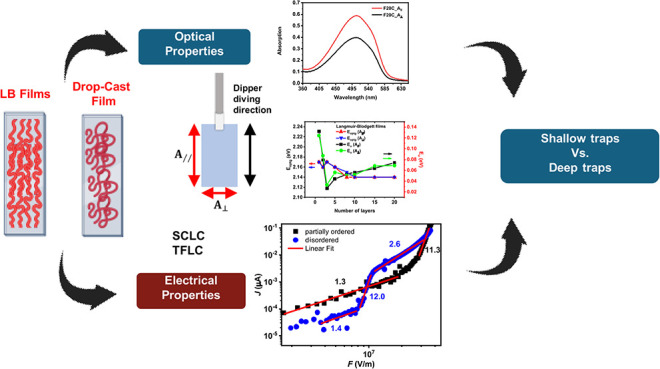

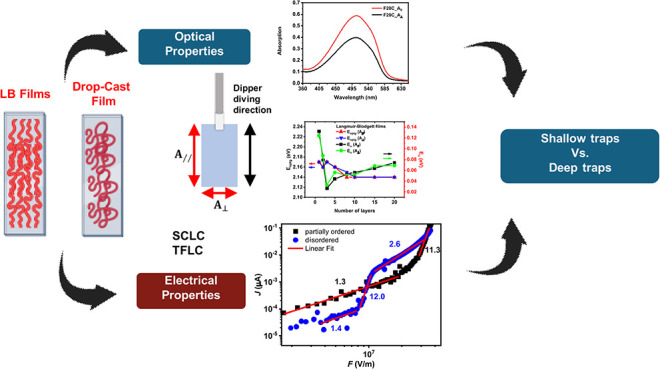

## 1 Introduction

Producing optoelectronic devices with high performance demands knowledge of band energies and gaps and, not less important, the states in the gap. Those states work as traps, reducing charge mobility or, even worse, working as recombination centers, that decrease the open-circuit voltage in solar cells.^1–4^ The processing methodology may induce the formation of different structures, which result in different states in the gap. In particular, devices produced from active molecules in solution possess, in the solid state, microstructures and morphologies inherited from the solution that directly affect the performance of the devices and the optoelectronic properties. In solution, several parameters directly affect the film in the solid state, such as solvent dielectric constant, evaporation rate, polymer solubility, deposition method (spin-coating, drop-casting, Langmuir–Blodgett, etc.), inducing inter- or intrachain interactions, and annealing after film deposition.^5,6^

In polymer semiconductors, the optical band gap gives information on the *π* → π* transition. Analyzing the dependence of the absorption coefficient *α*(*E*) close to the band edge, where the energy dependence can be described as^7^

1 $${\left(\alpha \left(E\right)h\nu \right)}^{1/\gamma }\propto A\left(h\nu -E_{optg}\right)$$

where *h* is Planck’s constant, *v* is the photon frequency, *E*_*optg*_ is the energy of the optical gap, and *A* is a constant related to the refractive index *n* and the effective mass of electrons and holes in the material. The exponent *γ* depends on the nature of the electronic transition and should be equal to 1/2 and 2 for direct and indirect transitions, respectively.^7–10^

The dependence of the absorption coefficient (eq 1) is obtained by considering band-to-band transitions in defect-free semiconductors, without taking excitons or band filling into account.^10^ Those factors may cause changes in the absorption band gap (and also subgap), impacting directly on eq 1. Organic semiconductors, like conjugated polymers, are excitonic, amorphous, and show complex subgap characteristics with the presence of traps.^4,10^ These characteristics give rise to long tails in the absorption band edge, which may overlap the fundamental transition and distort the *E*_*optg*_ determined using the Tauc plot method described in eq 1.^5,10^ Information on these subgap states is obtained directly from the spectra using the Urbach plot. Below the *E*_*optg*_, the spectral dependence of the absorption coefficient *α*(*hv*) with photon energy *hv* is known as Urbach’s empirical rule and is given by^1,4^

2 $$\alpha \left(E\right)\propto e^{\left(\frac{h\nu -E_{\mathrm{optg}}}{E_{u}}\right)}$$

where *E_u_* is the Urbach energy and characterizes the exponential decay of absorption. In a general way, in solids, the Urbach tail is associated with the subgap states as a result of thermal and structural disorder, or dynamic and static disorder, respectively.^7^ The contribution of both kinds of disorder is additive and results in the Urbach energy *E_u_*.^11,12^

Electrical properties are affected by the subgap states in different regimes. Defects observed in the band tail, related to Urbach energy, are classified as shallow traps. In the current density vs electric field (*J* vs *F*) behavior, these traps affect charge mobility and are observed in the low-field region, usually called the ohmic regime. In this way, *J* is proportional to *F* or *J* ∝ *F*. As the field increases, different regimes can be observed, generally following *J* ∝ *F^n^* with different values of *n*. In particular, for intermediate values of the field, we have space-charge-limited current (SCLC, *n* = 2) and trap-filled-limited current (TFLC, *n* > 2). The latter is characterized by the presence of an exponential distribution of deep traps. Finally, after these regimes, and once the deep traps are filled, the system again exhibits a space-charge-limited Current (SCLC), in this case trap-filled (again *n* = 2).^13^

The TFLC shows an exponential distribution of deep traps with energy *E* above the HOMO level:^14^

3 $$n_{t}\left(E\right)=\frac{N_{t}}{kT_{t}}\exp\left(-\frac{E}{kT_{t}}\right)$$

where *N_t_* is the trap density, and *T_t_* is a characteristic temperature associated with the energy distribution of traps. So, *E_t_* = *kT_t_* is the energy that characterizes the distribution of deep traps. The *n_t_* (*E*) distribution applied to the Poisson equation results in the following expression for the current density *J*:^13,14^

4 $$J=q^{1-l}\mu_{p}N_{\upsilon }{\left(\frac{2l+1}{l+1}\right)}^{l+1}{\left(\frac{l}{l+1}\frac{\epsilon_{0}\epsilon_{r}}{N_{t}}\right)}^{l}\frac{V^{l+1}}{L^{2l+1}}$$

where *μ*_p_ is hole mobility, *N_υ_* is the effective density of states in the HOMO orbital, *q* is the elementary charge, $l=\frac{T_{t}}{T}$ , and *ϵ*_0_,*ϵ*_r_ are the vacuum and relative dielectric constants, and *L* is the sample thickness.

Furthermore, the correlation of Urbach energy and electrical properties vs temperature and thickness has been the subject of study by different groups, in general, in inorganic systems like ZnO, perovskites, and chalcogenides.^3,4,12,15–17^ On the other hand, in organic semiconductors, the discussion seems to be limited, involving in particular solar cells, where the Urbach energy is related to the voltage loss. Unlike banded semiconductors, the Urbach energy in molecular semiconductors equals the thermal energy (*kT*) and is not related to energetic disorder. Static disorder determines the spectral broadening only near the absorption onset.^1,2,4^

Thus, there is a lack of works dealing with Urbach energy and film processing, the effect of thickness, and induced ordering, especially in organic semiconductors. Moreover, there is a complete lack of works dealing with ultrathin layers (under 100 nm thick), a region subject to interface effects. This work investigates film processing and its effect on the resulting optical and electrical properties. Because films and the active layer in devices are solution processed, we began evaluating solvatochromic effects in UV–vis absorption and emission. We then compare films prepared using different techniques, analyzing parameters, such as the Urbach tail and current-field characteristics in devices, and correlating them with subgap states and charge mobility. For MEH-PPV in solution, UV–vis spectra provide the optical gap (*E_optg_*) and Urbach energy (*E_u_*) while emission spectra yield the Huang–Rhys (***S***) and effective conjugation length (*n*). These parameters capture conformational, torsional, and solvent–polymer interactions that influence film formation. We further evaluate *E*_*optg*_ and *E_u_* in processed films using the Langmuir–Blodgett and drop-casting techniques (partially ordered vs disordered films), including polarized light to get information on different directions. In particular, the Langmuir–Blodgett technique allows us to analyze single-layer films into several layers. Finally, we correlate the optical Urbach energy with current density vs electric field in a diode-like device.

## 2 Experimental Section

MEH-PPV polymer (C_18_H_28_O_2_)_*n*_, with a molecular mass between 40,000 and 70,000, was purchased from Sigma-Aldrich. Glass slides, as substrates, were obtained from Precision Glass Line. ITO-coated glass substrates (8–12 Ω/sq and 100 nm thickness) were purchased from Delta Technologies. ITO substrates were cut into (13 × 18) mm^2^, and a central strip of (6 × 18) mm^2^ was protected with adhesive tape to define the ITO electrode area. The unprotected ITO region was removed using HCl (∼1 M) and zinc powder.

For substrate cleaning (glass or ITO), a two-step procedure was employed. The first step consisted of a multistage chemical process, with each stage performed for 20 min in an ultrasonic bath at 65 °C. The sequence was: (i) detergent with abundant water; (ii) a mixture of distilled water and detergent; (iii) acetone; and (iv) ethyl alcohol. The second step involved plasma cleaning under the following conditions: oxygen flux of 10 cm^3^/min, plasma exposure time of 10 min, frequency of 50 kHz, and pressure of 200 mTorr. The process was carried out using a Plasma Etch model PE-50.

The polymer was dissolved in monochlorobenzene (C_6_H_5_Cl) at a concentration of 0.5 mg/mL. Complete dissolution was achieved by stirring the solution with a magnetic stirrer for 20 h at ambient temperature. Film samples were deposited by using two different methods: Langmuir–Blodgett and drop-casting. For the Langmuir–Blodgett films, a KSV-2000 Langmuir trough model (Nima Technology) was used. A volume of 120 μL of the polymer solution was spread directly onto the water subphase. After solvent evaporation, the Langmuir film was compressed to the deposition pressure of 10 mN/m, corresponding to an area per monomer of 40 Å/monomer. The transfer to the substrate began after 10 min, while maintaining the surface pressure at 10 mN/m, to allow the film to stabilize and organize. The transfer ratio was monitored and maintained at (0.9 ± 0.3). More details about the Langmuir monolayer and the Langmuir–Blodgett films are given in the Supporting Information.

The drop-casting films were deposited by varying the spreading volume while maintaining the covered area, using increments of 25 μL from an initial value of 100 μL. The solvent was allowed to evaporate under saturated conditions over 24 hours. The samples, all prepared as described above, are listed in Table 1.

**Table 1:** Prepared Samples and Their Corresponding Codes^a^

Depositing method	Drop-casting (disordered)	Langmuir–Blodgett (partially ordered)
Sample code	C_100 μL	F1C – 1 layer
	C_125 μL	F2C – 2 layers
	C_150 μL	F3C – 3 layers
	C_175 μL	F4C – 4 layers
	C_200 μL	F5C – 5 layers
		F8C – 8 layers
		F10C – 10 layers
		F15C – 15 layers
		F20C – 20 layers

a For the drop-casting samples, the spread volume is indicated in the code (C_volume), while for the Langmuir-blogett samples, the number of layers is included in the code (F#of layersC). The solution concentration used in all casses was 0.5 mg/mL.

Absorption spectra were collected using a SHIMADZU UV-3600 Plus UV–visible spectrophotometer in the 280 to 760 nm spectral region. Emission spectra were obtained with a PC1-Photon Counting Spectrofluorimeter-ISS in the L-configuration under steady state, equipped with a xenon arc lamp as the excitation source. Data were acquired using an excitation wavelength of 400 nm, collecting emission spectra between 420 and 750 nm, with 1 mm slits and a 60° sample support angle. Solution spectra were recorded using a 1 mm quartz cuvette to minimize internal filter effects due to reabsorption during emission.

After the deposition of the disordered MEH-PPV layer onto ITO, to produce the devices, samples were placed in a thermal treatment chamber for 2 h at 50 °C under vacuum, resulting in a film thickness of (108 ± 10) nm. For the devices using LB films, the methodology described above was used to deposit a total of 21 layers, resulting in a film thickness of approximately (40 ± 5) nm. The top aluminum electrode was thermally evaporated at 6 × 10^–6^ mbar, resulting in a 100 nm thick aluminum layer. The effective device area was approximately (13.4 ± 0.6) mm^2^.

## 3 Results and Discussions

### 3.1 Monolayer Characteristics

Two different concentrations were tested to produce Langmuir monolayers: 1.0 and 0.5 mg/mL. Figure S1.a compares the isotherms for both concentrations and shows the reproducibility of the isotherms with 0.5 mg/mL. We spread 70 μL of the 1.0 mg/mL solution and 120 μL of the 0.5 mg/mLsolution onto the aqueous subphase using a microsyringe. The spreading solution was deposited drop by drop, avoiding the loss of material to the subphase. The lower area per monomer observed with the 1.0 mg/mL solution is a feature already observed by other authors^18,19^ and attributed to differences in conformational organization and aggregation at the air–water interface. A useful perspective is provided by the free-energy balance. The formation of an interfacial monolayer at the air–water interface competes with the thermodynamic (and kinetic) stability of aggregates already present in the spreading solution. Disaggregation carries a free-energy cost associated with breaking polymer–polymer interactions, as well as cooperative and conformational contributions. In parallel, organization at the interface involves changes in interfacial free energy and entropy, governed by polymer/water interactions and the ability of the chains to reorganize and minimize unfavorable contacts. In this case, the nonpolar polymer backbone at a highly polar water interface, exposing isolated chains to water, will be energetically costly. Under these conditions, preserving aggregates from the spreading solution at the interface is thermodynamically favored, opening the possibility of LB films to inherit aggregates from the spreading solution.

In general terms, the *π*–*A* isotherms have similar aspects to those shown by the authors.^18,19^ The inverse of the elastic modulus $C_{s}^{-1}$ was calculated from the Langmuir isotherm and is shown in Figure S1.b. At 10 mN/m, it is around $C_{s}^{-1}=16mN/m$ (or *C*_s_ = = 0.063 m/mN), close to the values determined by Saxena et al.^19^ Previous work on conjugated polymers like MEH-PPV indicates that high-boiling aromatic solvents, such as chlorobenzene, remain at the interface during Langmuir film formation, transiently plasticizing the monolayer and altering the surface pressure–area behavior compared to more volatile solvents, such as chloroform.^18,19^ In this way, the properties of the films prepared by the Langmuir–Blodgett technique are found to be very sensitive to processing conditions such as solvent and solution concentration, barrier speed, and spreading solvent, and therefore play an important role in determining the film morphology and performance of the resulting devices. Figure S1.c shows monolayer stabilization. The barrier speed was set to 5 mm/min during the isotherm and film stabilization. After 30 min at constant *π* = 10 mN/m, the change in area is around 1 Å^2^, achieving a maximum of 2 Å^2^ after 2 h (green dashed lines). We considered 30 min as 50% of the relaxation. The complete procedure before initiating film transfer was around 50 min: spread the solution onto the subphase, wait 10 min to allow solvent evaporation, initiate compression, and then wait an extra 30 min for film relaxation. The transfer started with the substrate immersed in the subphase and used an upper and down stroke at 5 mm/min to achieve a transfer rate of (0.9 ± 0.3). Film quality can be validated with the AFM image of the LB film shown in Figure S1.d. The three-layer thickness film shown in Figure S1.d has a homogeneous and compact morphology.

### 3.2 MEH-PPV in Solution

Conjugated polymers in solution exhibit complex structures that give rise to different backbone and side-chain conformations, including broken conjugation caused by torsional disorder, intermolecular interactions, and aggregation. To evaluate the solvatochromic effects of the solvent, measurements were performed in solution. A starting solution with a concentration of 0.0033 mg/mL was prepared, and successive 2 μL aliquots of a stock solution at 0.5 mg/mL were added.^20^ Figure 1a shows the absorption spectra for MEH-PPV in solution, with a maximum at 506 nm. The effect of concentration on the polymer structure in the chosen solvent was monitored through the optical band gap energy (Figure 1b and c). For organic semiconductors, this energy is typically obtained from the optical absorption edge energy and corresponds to the energy required to generate an excited state.^8,9^

**Figure 1:**
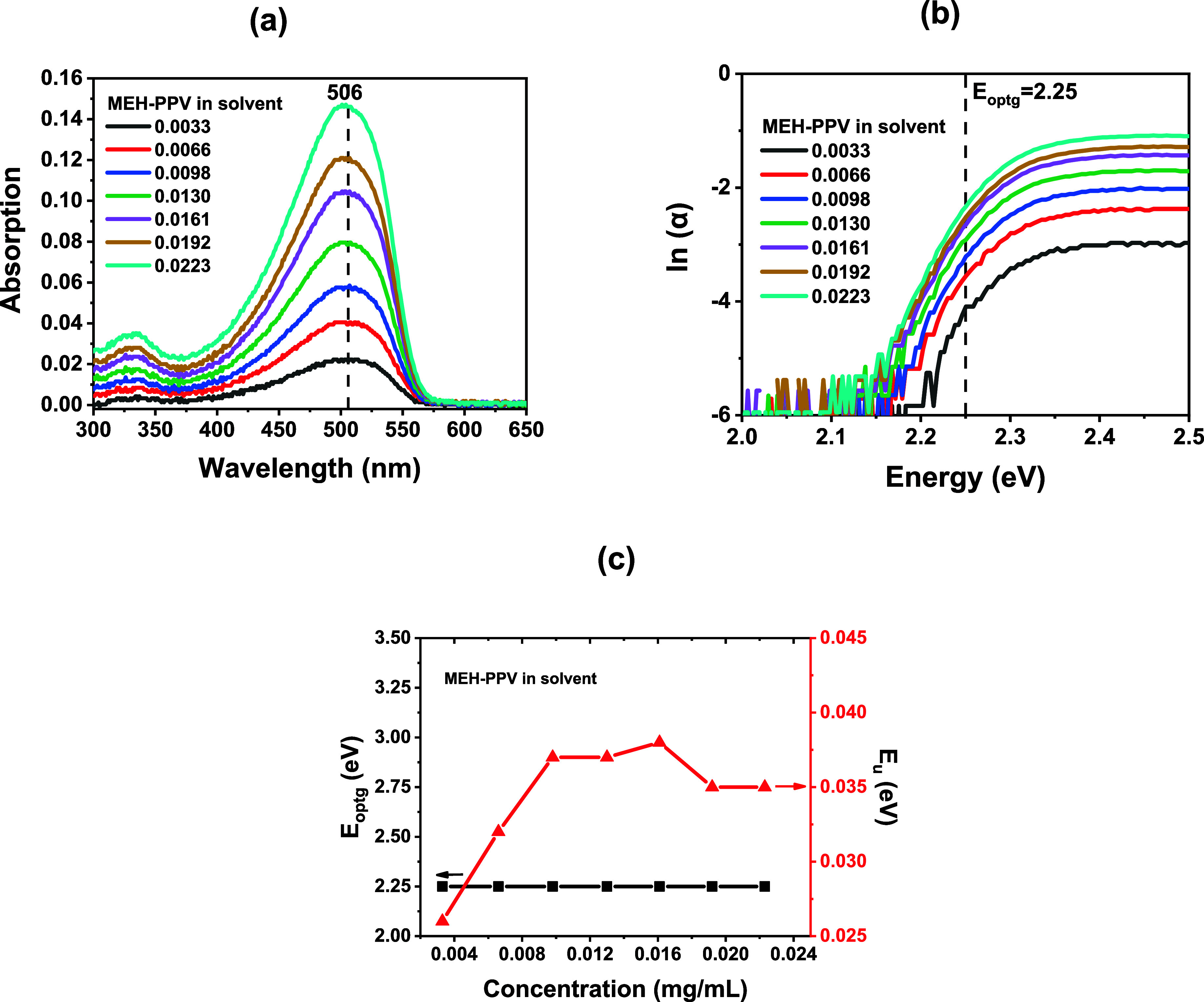
(a) Absorption spectra of MEH-PPV in solution. Concentrations were varied between 0.0033 and 0.0223 mg/mL. (b) Urbach plots ln­(*α*) ×Energy (eV). The *E*_*optg*_ is highlighted with a dashed line. Concentrations are indicated in mg/mL. (c) Optical band gap and Urbach energy vs concentration.

Equation 1 was applied to obtain the value of *E*_*optg*_ for all concentrations. This analysis, optical band gap vs concentration (Figure 1.c), may elucidate the formation of specific conformations and aggregates that, subsequently, are transferred to the solid state in the form of films.^5^ Solvent and solvent-chromophore properties, such as dielectric constant, refractive index, van der Waals interactions involving side groups, and polymer solvation, influence the *π* and π* orbitals, leading to changes in the *π* → π* transition and consequently changes in the optical behavior.^21^ In other words, an abrupt change in the aggregation state of the polymer, resulting from chain collapse due to strong intermolecular interactions, may produce measurable variations in the optical bandgap. In the present case, the obtained value of *E*_*optg*_ = 2.25 eV remained constant across all analyzed concentrations, indicating no change in the aggregation state.

Even in solution, the spectra exhibit a long exponential tail for energies below the *E*_*optg*_. We suggest applying similar Urbach analyses for the MEH-PPV in solvent. In this case, the conformational complexity of the polymer is expected to produce comparable effects in the absorption spectra, leading to the emergence of band tails. Thus, we propose that distinct processes may affect the conjugated backbone, associated with the *π* → π* electronic transition, and the band tail, which is more susceptible to conformational and side-chain interactions. Figure 1c shows *E_u_* as a function of concentration, displaying a linear increase at lower concentrations, with values of *E*_u_ = 0.026 eV at *C* = 0.0033 mg/mL and *E_u_* = 0.038 eV at *C* = 0.0161 mg/mL. At higher concentrations, however, it decreases to 0.035 eV, suggesting a possible stabilization. These variations may arise from structural and conformational changes induced by increasing the concentration. Conjugated polymers are subjected to side-chain and solvent interactions that influence their final structure and conformation.^5^ As the concentration increases, a large number of conjugated chains are present in the solution, promoting intermolecular organization, which leads to stabilization of the *E_u_* for the concentrations *C* ≥ 0.0192 mg/mL.

The optical band gap energy is strongly affected when only a few monomers contribute to the conjugation length. However, once a sufficiently large number of monomers are involved, small variations (e.g., the addition or removal of one or two monomers) have only a minor impact.^22,23^ The results shown in Figure 1c are consistent with this behavior: no detectable change in the optical gap is observed despite variations in the band tail, indicating increased side-chain interactions. The microstructure changes may be affecting the morphologies of thin films produced from such polymeric solutions, with direct implications for device performance.^5,6^

When analyzing the emission processes, it is important to take into account energy transfer from short conjugation segments to longer segments. It is highly efficient, and as a consequence, the molecular assembly governing emission differs from those controlling absorption.^6,20^ A key feature of MEH-PPV emission spectra is the presence of well-resolved vibronic replicas. Observing these replicas clearly is an experimental advantage, as it provides access to additional structural and photophysical information. Following the definitions in references 24,25 , the Huang–Rhys parameter relates the intensity of the transitions between the fundamental vibronic level of the excited state and the vibronic level of the fundamental electronic state:

5 $$I_{mj}=\frac{e^{-S_{j}}{\left(S_{j}\right)}^{m}}{m!}$$

where *j* = 0,1,2,3,··· are the different vibronic modes in the excited electronic state, and *m* = 0,1,2,3,··· are the different vibronic modes in the fundamental electronic state. *S_j_* is the Huang–Rhys factor for the vibrational mode *j*. Relating the intensities for *m* = 1 and *m* = 0, we can get *S_j_*:

6 $$S_{j}=S=\frac{I_{\left(1j\right)}}{I_{\left(0j\right)}}$$

where *I*_(0*j*)_ and *I*_(1*j*)_ are the maximum intensities in the photoluminescence spectra due to the pure electronic transition (00) and the first vibronic band (01).

According to Yu et al.,^26^ ***S*** can be related to the effective conjugation length by the following equation:

7 $$S=ae^{\left(-n^{2}/b\right)}$$

where *n* is the conjugation length, and *a* and *b* are empirical parameters. Here, we used 3.2 and 38, respectively (values taken from references 24,26 ).

Therefore, to investigate the contributions of structural disorder to the emission spectra, we analyze the emission by determining the position, area, and relative intensity of the vibronic bands. Gaussians are used to extract these parameters. Figure 2a shows the emission spectra for the same concentration presented in Figure 1. The pure electronic transition and the first and second vibronic bands are labeled 00, 01, and 02, respectively.

**Figure 2:**
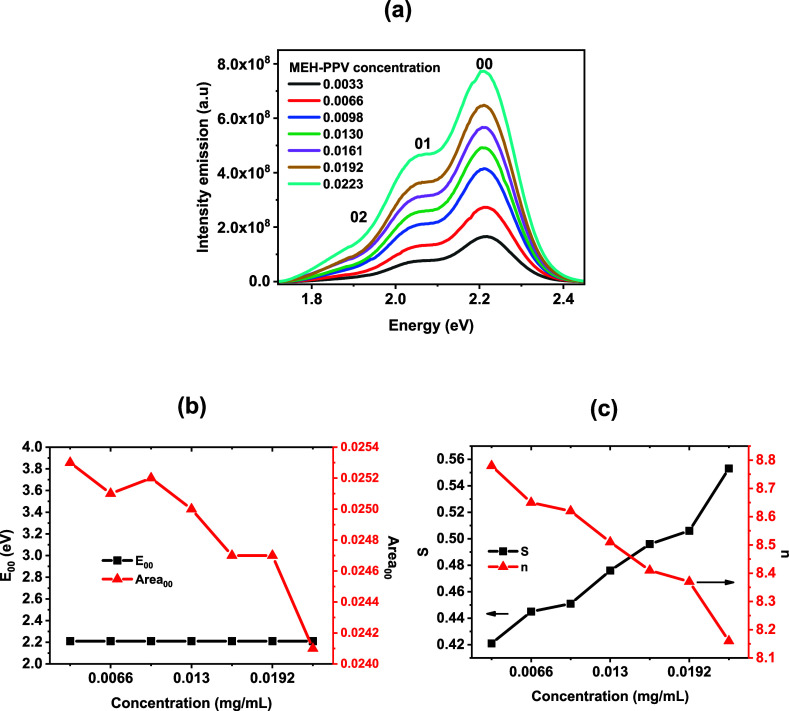
(a) Emission spectra of MEH-PPV in chlorobenzene at different concentrations. (b) Energy and area of the 0–0 (pure electronic) transition as a function of concentration. (c) Huang–Rhys factor (***S***) and effective conjugation length (*n*) as a function of concentration.

Since the position and area of the 00 pure electronic transition are shown in Figure 2b, the energy position of the first vibronic band remains essentially unchanged with concentration, whereas the area decreases almost linearly. As seen in Table S2 of the supporting material, the relative intensity of the 00 transition also decreases with concentration, indicating enhanced nonradiative relaxation and reduced emission efficiency. This effect suggests the presence of intermolecular interactions that are not captured by the analysis of the optical band gap. To quantify those conformational and structural changes, we use the Huang–Rhys parameter ***S*** (eq 7), obtained through spectral analysis of the spectra shown in Figure 2a using a Gaussian function. This parameter provides insight into the coupling between the exciton and the vibrational modes of the conjugated backbone, and it can be used to infer an effective conjugation length.^24^ The dependence of ***S*** and *n* with concentration is shown in Figure 2c. It is evident that ***S*** increases while *n* decreases, exhibiting opposite behavior with the concentration increase. ***S*** rises from 0.421 at *C* = 0.0033 mg/mL to 0.553 at *C* = 0.0223 mg/mL. Variations in ***S*** are associated with the number of molecular configurations that enable relaxation of the excited state into different vibrational levels in the ground electronic state. From an energetic perspective, an increase in ***S*** corresponds to a larger portion of the excitation energy being dissipated into vibrational modes, i.e., increased vibrational energy loss.

Nevertheless, changes in *n* are not significant; the mean conjugation length continues to be around 8, as predicted.^27,28^ Due to energy transfer, emission originates from conjugated segments that are different from those responsible for absorption. The results indicate that the emitting segments exhibit lower energetic disorder, as evidenced by the small variations in the ***S***, *n*, the energy position of the vibronic replicas, and the areas of the corresponding Gaussian components. From this, we infer that species with higher energetic disorder contribute only weakly to the emission. Only a small fraction of aggregated species contributes to the observed decrease in relative intensity and area of the 00 vibronic band.

### 3.3 Film Characterization

Films were produced from the solution characterized in the preceding section. The absorption spectra of the LB films with different numbers of layers are shown in Figure 3a. The absorption at *λ* = 515 nm was used to monitor the film growth, as presented in Figure 3b. As additional layers are deposited, the absorbance at 515 nm increases linearly with the number of layers. In this case, the absorption growth rate is 0.023 per layer.

**Figure 3:**
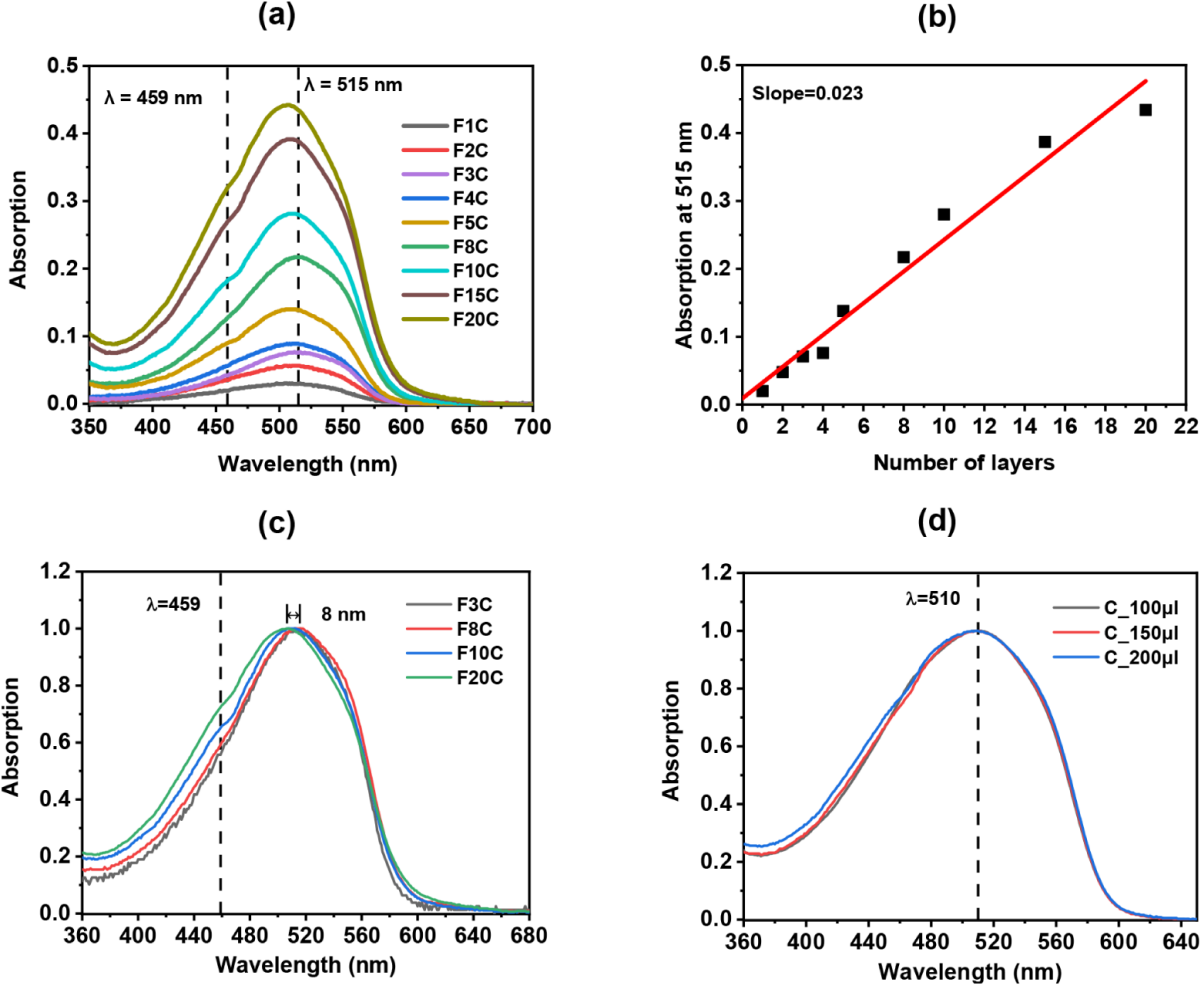
(a) Absorption spectra of MEH-PPV LB films with different numbers of layers. (b) Absorption at 515 nm as a function of the number of layers. The red line shows the linear fit, yielding a growth rate of 0.023 absorbance per layer. (c) Normalized absorption spectra of LB films highlighting the shift in the maximum absorption. (d) Absorption spectra of drop-casting films, showing a maximum at 510 nm.

Figure 3c shows the normalized absorption spectra of the LB films, highlighting an 8 nm shift in the absorption maximum as additional layers are deposited. For comparison, Figure 3d displays the spectra of the drop-casting films, where no shift is observed, and the maximum remains at 510 nm. In contrast, the maximum absorbance of the LB films shifts gradually, from 515 nm for the single-layer film to 507 nm for the 20-layer film (Figure 3a). The higher absorption maximum observed for films with a low number of layers is consistent with the well-known trend that LB films exhibit a higher effective conjugation length compared to drop-casting.^19,29,30^ The blue shift observed in LB films as the number of layers increases is associated with structural organization induced by the deposition method,^31^ in which *π* → *π* stacking may be favored. This behavior may be related to several factors, including aggregation, polydispersity, chain folding, and the transition from interface-dominated effects to bulk-like effects as the film becomes thicker.^32,33^

Starting from 10 layers, a shoulder at 459 nm (Figure 3a) becomes evident in the spectra. This feature is the characteristic signature of the blue phase of MEH-PPV.^31,34^ The emergence of this signature as the film grows is consistent with the transition from interface-dominated behavior to bulk-like behavior, indicating the formation of regions with different local order and distinct conjugation lengths.

Considering its conjugated backbone, MEH-PPV behaves as a uniaxial material and therefore exhibits different absorption responses depending on the polarization direction of the incident light and the degree of film organization. The polarized UV–vis spectra for both LB and drop-cast films are presented in Figure 4, and the polarization and dipping directions used for the LB films are illustrated schematically in Figure 4a. Polarized UV–vis measurements were performed to investigate the molecular ordering induced by the LB deposition method in comparison with drop-casting. Because the UV–vis absorption of conjugated polymer segments is highly sensitive to the polarization direction of the incoming light, Figure 4 reveals a clear anisotropic response in the LB films, whereas the drop-cast films behave isotropically. For the LB samples (Figure 4b and c), the absorption intensity is higher when the spectra are recorded with light polarized parallel to the dipping direction (Figure 4b and c), consistent with the orientation indicated in the schematic of Figure 4a.

**Figure 4:**
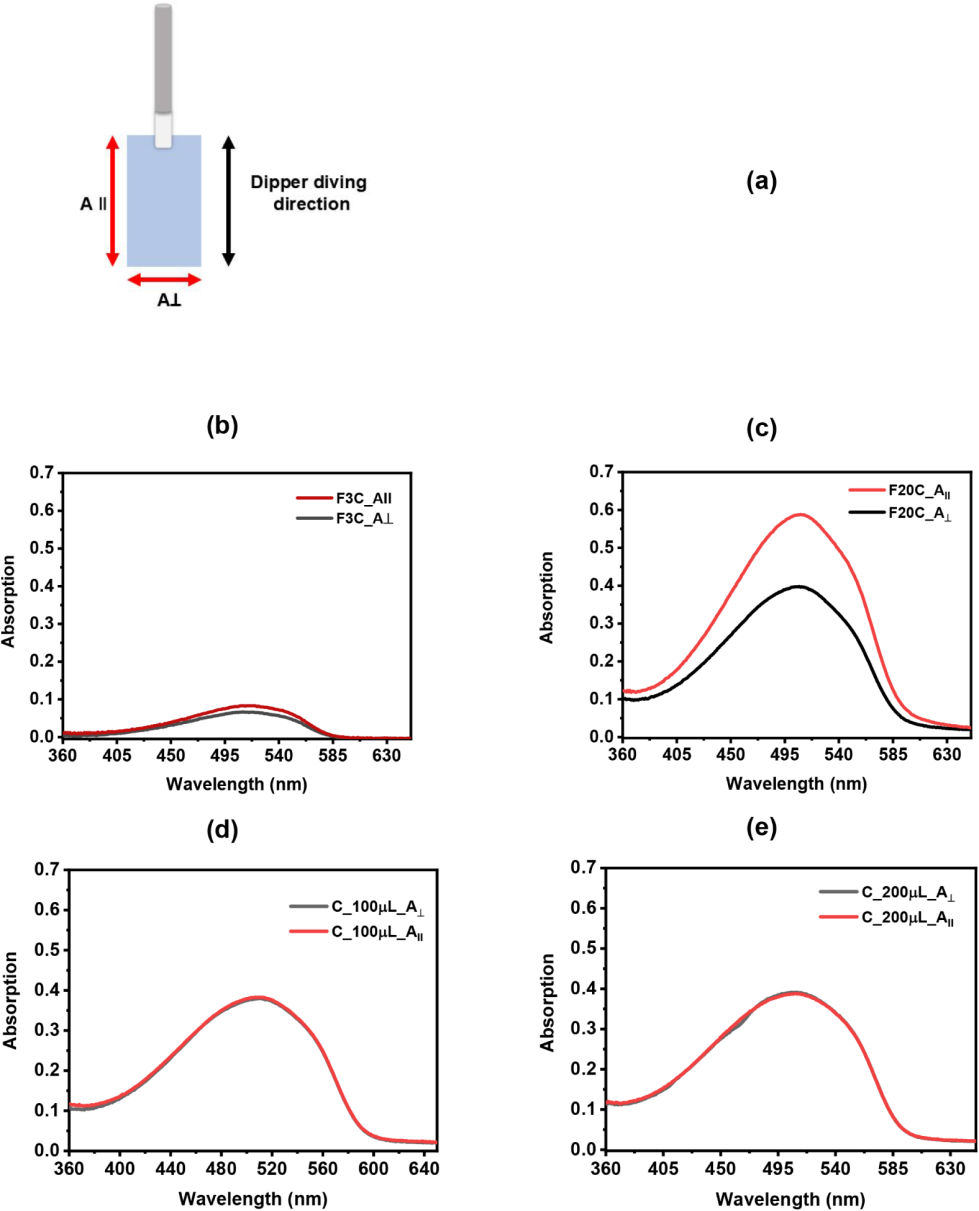
(a) Schematic illustration of the dipping direction and light polarization: *A*_∥_ and *A*_⊥_ refer to absorbance measured with light polarized parallel or perpendicular to the dipping direction, respectively. Polarized UV–vis spectra of LB films for three (b) and 20 layers (c). Polarized UV–vis spectra of drop-cast films prepared with (d) 100 and (e) 200 μL of solution.

To estimate the degree of ordering in the LB films, we use the dichroic difference, Δ*A* = *A*_∥_ – *A*_⊥_ at maximum absorption.^35,36^ Figure 5 summarizes the dichroic difference for the LB films with different numbers of layers. The value increases slowly from close to zero (0.002 for the monolayer) to 0.223 for 15 layers, followed by a slight decrease to 0.191 for 20 layers. The fact that Δ*A* > 0 for all samples indicates that the conjugated backbones are preferentially oriented along the dipping direction. Another important aspect is that the shoulder at 459 nm observed in nonpolarized spectra (Figure 3a) does not appear in the polarized spectra (Figure 4). This suggests that the group of backbones that produce this feature is oriented along a direction that does not coincide with either the parallel or the perpendicular axes relative to the dipping direction. When polarized light is used, only the polymer backbones that are preferentially oriented along the polarization direction contribute more strongly to the absorption. This behavior reflects the presence of molecular domains with different orientations within the film.

**Figure 5:**
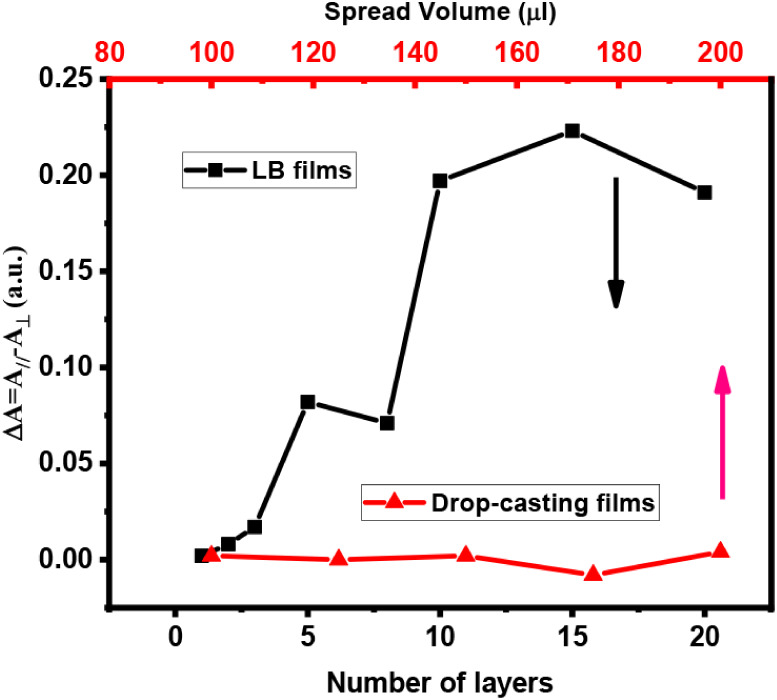
The dichroic difference is determined at the maximum of each spectrum. *A*_∥_ and *A*_⊥_ are measured using polarizers parallel and perpendicular to the dipping direction, respectively.

The results in Figure 5 show that the LB technique produced films with preferential orientation of the conjugated backbones along the dipping direction. In contrast, the drop-casting films show no preferential optical direction. For drop-cast films, the dichroic difference remains at the detection limit, oscillating between positive and negative values. In contrast, LB films show a consistent increase in dichroic difference up to 15 layers. This behavior is a consequence of the forces acting at the air/water interface during LB deposition, which promote more efficient molecular organization and modify the polymer conformation relative to that in solution. Another important aspect is that MEH-PPV at the air/water interface forms regions with different microstructures compared to drop-cast films, leading to differences in both *E_u_* and *E*_*optg*_.

### 3.4 *E_u_* and *E_optg_* Behavior Relative to Thickness

According to eqs 1 and eq 2, we obtained *E*_*optg*_ and *E_u_* for the films produced by the different deposition methods. Figure 6a and b shows the results, revealing distinct trends for each parameter depending on the deposition method.

**Figure 6:**
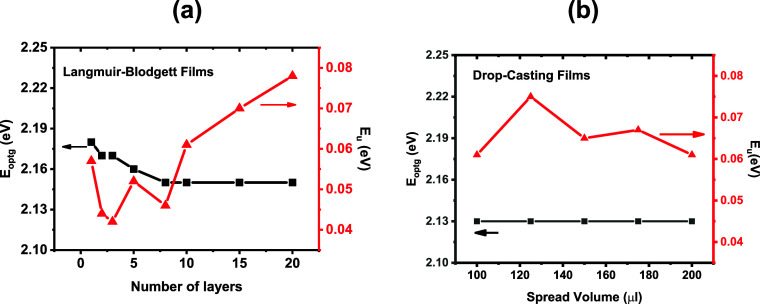
*E*_*optg*_ and *E_u_*: (a) LB films with different numbers of layers and (b) drop-casting films with different solution volumes.

The LB films exhibit two distinct regimes. For 1 to 8 layers (Figure 6a), *E*_*optg*_ decreases from 2.18 eV to 2.15 eV,while *E_u_* shows no well-defined trend. Starting at 10 layers, the *E*_*optg*_ stabilizes at 2.15 eV, and *E_u_* begins to increase consistently, rising from 0.061 eV at 10 layers to 0.078 eV at 20 layers. In contrast, the drop-casting films (Figure 6b) display a constant *E*_*optg*_ = 2.13 eV for all volumes, with only small variations in *E_u_.* The differences observed in the values of *E_u_* and *E_optg_* for each technique are linked to two previously discussed factors: interfacial effects (substrate/polymer and polymer/polymer interactions) and differences in molecular packing, particularly π-stacking. When comparing these values obtained from the absorption spectra of the solutions, it becomes evident that the solution spectra are narrower, well-defined, and show no shoulders, in contrast with the films. This affects the determination of both *E*_*optg*_ and *E_u_*. In solution, *E*_*optg*_ = 2.25 eV for all concentrations, indicating systems with relatively homogeneous conjugation lengths and homogeneous sizes. In contrast, films presented more heterogeneous systems, which is reflected in the broad absorption band. Furthermore, *E_u_* shows lower values in solution than in films (see Figures 1c and 6) indicating a strong influence of solid-state packing. Since the dynamic component of *E_u_* should remain similar, the increase in *E_u_* in the films reflects changes in the static disorder introduced by the processing method. This structural disorder has a significant impact on *E*_*optg*_, which showed values at least 10 meV, higher values in solvent than in films.

### 3.5 *E*_*u*_ and *E*_*optg*_ Behaviors with Orientation

In the preceding section, we discussed the optical gap and Urbach energy without considering the preferential orientation of the LB films. The analysis, therefore, focused on thickness-dependent vs bulk effects. An intriguing question now arises: how does the molecular ordering induced by the deposition method, combined with the polarization direction of the incident light, influence both *E*_*optg*_ and *E_u_*. These parameters are summarized in Figure 7.

**Figure 7:**
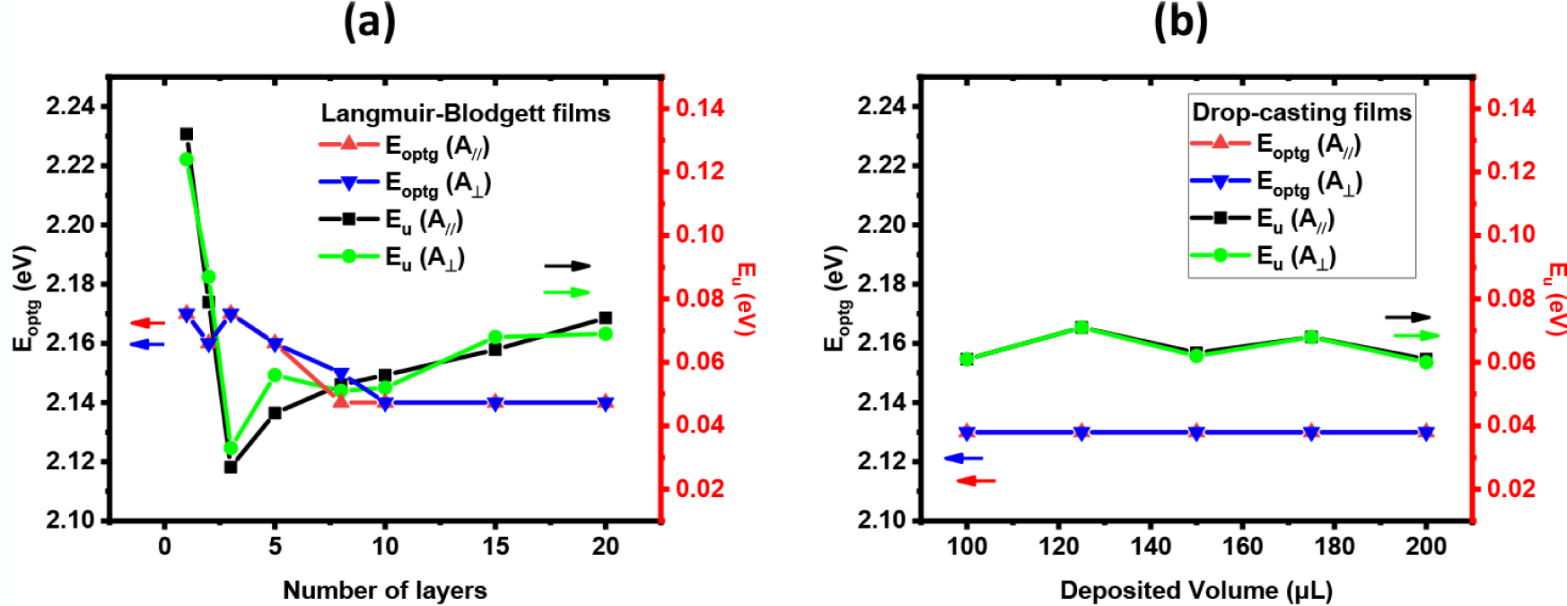
Directional analysis of the optical gap and Urbach energy for (a) LB films and (b) drop-casting films. The LB films show a clear dependence on the number of layers for both parameters; however, the dependence on polarization direction is subtle. In contrast, the drop-casting films show no dependence on either direction or spread volume.

The LB films (Figure 7a) continue to exhibit two distinct behaviors. For the smallest number of layers, *E*_*optg*_ decreases for both polarizations, from 2.17 eV for F1C_∥ and F1C_⊥ to 2.15 eV for F8C_⊥ and 2.14 eV for F8C_∥. In this regime, *E_u_* displays values higher than those obtained from the nonpolarized: 0.124 eV for F1C_⊥ and 0.132 eV for F1C_∥, compared to 0.057 eV for F1C. For a larger number of layers, *E*_*optg*_ stabilizes at 2.14 eV and *E_u_* increases with layer number. However, the polarized *E_u_* values remain lower than their nonpolarized counterparts, for example, 0.052 eV (F10C_⊥) and 0.056 eV (F10C_∥) compared to 0.061 eV for the nonpolarized F10*C*. This behavior is observed for up to 20 layers. The drop-cast films show *E*_*optg*_ values identical to those obtained under the nonpolarized light, 2.13 eV, independent of the deposited volume. Similarly, *E_u_* exhibits values very close to the nonpolarized light (Figure 7b), confirming the polarization direction-independent character of these films.

An intriguing observation is that *E_u_* exhibits very similar values in both polarization directions; the difference between the values obtained in the parallel and perpendicular directions oscillates between positive and negative as the number of layers increases. If we assume that both directions share the same dynamical contributions (temperature contribution), then any difference in Urbach energy arises solely from structural disorder. However, not all polymer backbones are perfectly aligned along the preferred dipping direction. When polarized light is used, only the segments oriented along the polarization direction contribute more strongly to the absorption. In other words, the structural defects responsible for *E_u_* appear to be similarly distributed in both directions, resulting in nearly identical Urbach energies.

It is important to highlight that the asymptotic *E_u_* value observed for the LB films is similar to that obtained for drop-casting films. Overall, the results indicate that the molecular organization in these systems is highly complex, linked, and influenced by multiple factors, including defects, polydispersity, aggregation, and the transitions from interface-dominated to bulk-dominated behavior.^32,33^ According to Figure 7, the transition from interface to bulk probably occurs at approximately 10 monolayers.

### 3.6 Emission of LB and Drop-Casting Films

Using the emission spectra of films, we identified the vibronic replicas in the same way as in solution and assigned the same labels to the pure electronic transition (00) and to the first (01) and second (02) vibronic replicas. Table 2 shows the Huang–Rhys factor ***S***, the mean conjugation length *n,* and the difference in energy between 00 and 01 transitions for the different polarizer configurations.

**Table 2:** Huang–Rhys Parameter (***S***) and Mean Conjugation Length (*n*) Determined from the Emission Spectra of LB (8 Layers) and Drop-Cast Films^a^

Excitation beam polarizer	Emission beam polarizer	***S***	*n*	*E*_00_ – *E*_01_ (meV)
**F8C**				
Without polarizers		0.406	8.85	134
90°	90°	0.450	8.63	151
90°	0°	0.542	8.21	148
0°	90°	0.542	8.21	154
0°	0°	0.160	10.66	97
**C_100 μL**				
Without polarizers		0.493	8.43	158
90°	90°	0.553	8.17	159
90°	0°	0.575	8.08	155
0°	90°	0.550	8.18	162
0°	0°	0.486	8.47	156

a The polarizer angles used for excitation and emission are indicated.

From the data shown in Table 2, it is important to distinguish the possible measurement configurations: the first column refers to the polarizer in the excitation beam, and the second column refers to the polarizer in the emission beam. Two configurations deserve particular attention: parallel polarizers (both oriented in the same direction, 0°0° or 90°90°) and cross polarizers (0°90° or 90°0°). In the parallel configuration, the backbones are excited, and their emission is detected along the same direction. In the crossed configuration, excitation occurs along one direction while emission is detected in the perpendicular direction, which is possible due to the efficient energy transfer between molecules. Without polarizers, we obtain average values for the parameters ***S***, *n*, and energy difference *E*_00_ – *E*_01_. When polarizers are included, it becomes essential to highlight the results obtained under parallel and cross configurations, as they provide directional sensitivity to the emission process. It was observed that the ***S*** parameter for the 0°0° orientation of the F8C film is lower than in the other configurations. According to the literature^24,37^ segments with longer conjugation lengths tend to exhibit lower *S* values. In our results, the mean conjugation length *n* shows similar values for the 90°90° configuration and for the crossed polarizer measurements, but increases by approximately two units in the 0°0° configuration, where the excitation and emission polarizers are aligned parallel to the dipping direction. This behavior suggests that the dipper movement, influenced by the meniscus at the substrate/water interface, induces the deposition of polymer chains with longer conjugation lengths preferentially along that direction. However, this slight change in the mean conjugation length (from 8 to 10) is not reflected in the *E*_*optg*_ due to the asymptotic behavior of this parameter with the number of monomers.^22,23^

From the Gaussian fitting in the emission spectra, we determined the energy difference *E*_00_ – *E*_01_. For the drop-casting films, this separation decreases with increasing deposited volume and shows no significant variation among the different polarizer configurations. The LB film (F8C) showed a lower separation between the pure electronic emission and the vibronic one, with the lowest value obtained for the 0°0° configuration.

To investigate the vibrational modes of MEH-PPV, Raman measurements were performed on the LB films. The spectra were acquired at room temperature over the spectral range of 900–1700 cm^–1^ and are presented in Figure S2 of supporting material, highlighting the main spectral contributions.

The band at 964 cm^–1^ is attributed to the out-of-plane bending mode of the C–H in the vinyl group; the band at 1109 cm^–1^ corresponds to the C–C stretching coupled to the C–H twisting of the phenyl group; the peak at 1309 cm^–1^ is assigned to the C=C stretching coupled to the C–H twist in the vinyl group. The 1557 cm^–1^ mode appears as a shoulder of the 1582 cm^–1^ band, both mainly associated with the phenyl ring stretching vibration. Finally, the band at 1622 cm^–1^ is attributed primarily to the C–C stretching of the vinyl group.^24,38–41^ Table 3 shows the energy values of the different vibrational modes.

**Table 3:** Vibrational Modes Observed in Raman Spectra and the Corresponding Energy

Vibrational modes	Spectral position (cm^–1^)	Energy (meV)
C–C stretching coupled to a phenyl ring twist	1109	137.5
C–C inter-ring stretching	1282	159.0
C=C stretching coupled to a C–H twist of the vinyl group	1309	162.3
C=C stretching of the phenyl ring	1582	196.0

It is well established in the literature^24,26^ that the vibronic feature (01) in the emission spectra arises from several vibrational modes that are strongly coupled to the electronic transitions. Raman spectroscopy of the material provides information about the vibrational modes that may be activated within the energy range of the vibronic structure.^24,38,39^ At high temperatures (*T* > 300 K), such as room temperature, the 01 contribution appears as a broad band separated from the 00 transition by approximately 142 meV. In temperature-dependent experiments, this energy separation is known to shift,^24,38^ and it correlates well with the vibrational mode at 1113 cm^–1^ (≈138 meV), given their similar energies.

Table 3 shows the vibrational-coupling energy differences (*E*_00_ – *E*_01_) extracted from the emission spectra of the films. For the drop-cast films, they are closest to the vibrational mode at 1309 cm^–1^ (162.3 meV). In contrast, for the LB film, the energy separation is closer to the vibrational mode at 1109 cm^–1^ (137.5 meV). The energies of the vibrational modes that may contribute to vibronic coupling are listed in Table 3.

The energy of the 01 vibronic contribution depends on the electronic localization length and can, therefore, vary with structural order, temperature, and polarization. These factors allow the exciton to probe different electron–phonon interactions, resulting in energetic variations of the vibronic contribution.^20,24,38^ When polarized light is used, the photoluminescence spectrum may change its relative vibronic contributions, reflecting variations in the vibrational modes associated with the electronic transition. Such changes arise from differences in molecular organization within the sample that interact differently with the polarized excitation.^24,38,42,43^

We observed that for the F8C LB film in the 0°0° configuration, the value of *E*_00_ – *E*_01_ differs significantly from the other polarization configurations, yielding 97 meV. This indicates that a different vibrational mode is being coupled in this orientation. As mentioned earlier, different chromophores, with distinct conjugation lengths and conformational structures, tend to exhibit different electron–phonon interaction intensities. This is because excitation-energy transfer between chromophores can lead to different relaxation pathways, and these pathways depend on the polymer’s conformational structure.

In this context, the vibrational mode whose energy most closely matches the observed variation in the 01 vibronic contribution is the 964 cm^–1^ mode (≈119 meV), assigned to out-of-plane C–H bending of the vinyl group. This vibration originates from the dihedral angle between adjacent monomeric units and is typically forbidden in the Raman spectrum of a fully planar polymer configuration.^24,40,41^ The comparison between LB and drop-cast films highlights that the deposition method induces different structural organizations for the chromophores responsible for the emission. In particular, the 0°0° configuration of the LB film (F8C) shows that the use of polarized light alters the relative contributions to the 01 vibronic band, indicating that a different vibrational mode becomes dominant in shaping the band profile. This reinforces the idea that the LB process produces anisotropic structures with conjugation lengths aligned along the dipping direction. When compared with the solution, the films generally show higher values of *n*, except for drop-cast films prepared with the largest volumes, in which disorder effects dominate. Altogether, the results from both solution and solid-state measurements provide consistent evidence for the role of aggregation and conformational disorder in determining the effective conjugation lengths and vibronic structure of MEH-PPV

### 3.7 Current Density *J* vs Field *F* of Diode Devices

To investigate the distribution of charge traps, diode-type devices were fabricated by using the previously described deposition techniques, allowing control over the degree of structural order in the active layer. The current curves as a function of electric field (*J* vs *F*) for these devices are presented in Figure 8.

**Figure 8:**
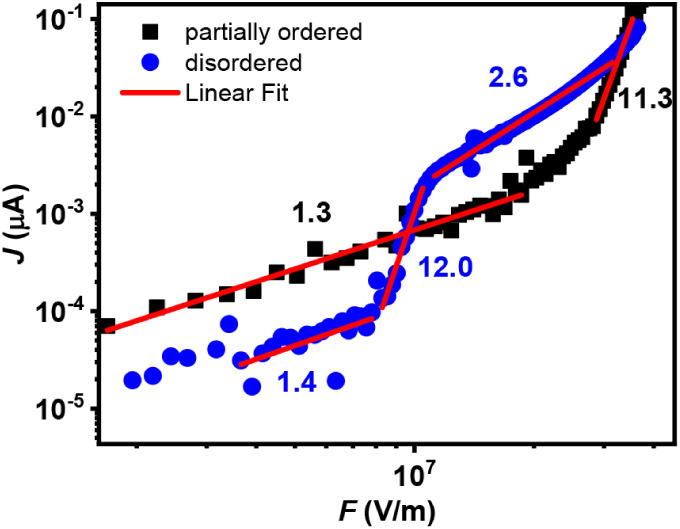
Log–log plots of *J* vs *F*, characteristic of the partially ordered and disordered active layer devices.

The log vs log plot of *J* vs *F* in Figure 8 highlights the different transport regions. Linear fitting in each region yields the slope *n* of the relation log­(*J*) ∝ *n* · log­(*F*), which is used to identify the dominant mechanism (values of *n* are indicated in Figure 8). At low field, both devices show slopes close to one (*n* = 1.3 for the disordered active layer and *n* = 1.4 for LB), indicating behavior close to the ohmic regime. This suggests an efficient charge injection from the ITO electrode in both cases. The slight deviation from ideal ohmic behavior (*n* = 1) is attributed to the small energy mismatch between the ITO work function and the polymer HOMO level. Even so, the current density of the LB device, below 10^7^ V/m, is higher than that of the disordered active layer device, indicating a lower density of shallow traps or reduced injection barriers. Details on the statistical analysis to determine exponents are provided in the Supporting Information.

For the disordered active layer device, the low-field region yields *n* = 1.4. Nevertheless, it is important to note that the ohmic regime extends over a field range much narrower than that in the LB device. In other words, the transition to the next transport regime (with a different *n* value) occurs at lower electric fields. Additionally, the disordered active layer device exhibits strong current fluctuations at low fields, which increase the uncertainty in the fitted slope.

These instabilities may be associated with discontinuities, surface traps, or internal barriers arising from the less controlled morphology of the disordered film. After the initial regime close to ohmic behavior, both devices show a sharp increase in current density, with *n* values greater than 2, characteristic of the trap-filled limit (TFLC) regime. This regime indicates the presence of an exponential distribution of deep traps. As shown in Figure 8, the disordered device show *n* = 12.0, while the LB shows *n* = 11.3. According to eqs 3 and 4, these correspond to characteristic trap energy *E_t_* of 0.292 and 0.276 eV, respectively. Thus, the energy associated with the exponential trap distribution is slightly lower in the LB film. The earlier onset of this region in the drop-casting device indicates a lower total trap density compared with the LB device. The density of trap states can be determined from the crossover voltage *V_TFLC_* between TFLC behavior and the trap-filled region^13,14^

8 $$V_{TFLC}=\frac{qH_{t}L^{2}}{2\varepsilon_{0}\varepsilon_{r}}$$

Using eq 8, we determined the trap density, *H_t_* = 3.33 × 10^16^ cm^–3^ for the drop-cast device, and estimated *H_t_* = 3.94 × 10^17^ cm^–3^ for the LB device. These values can be compared with the work by Chu et al.,^44^ where they found values around 10^18^ cm^–3^ for samples with 50 and 100 nm thickness, respectively. Differences may be attributed to film processing and different organic materials. Nevertheless, we found a higher density of traps in the LB film than in the drop-cast. In other words, the LB film contains a higher density of traps, but these traps are distributed over a narrower energy range.

Thus, the comparative analysis reveals that although the LB and SC devices present similar exponents at low voltage, only the LB film demonstrated relatively more stable charge transport over the entire operating range, with coherent transitions between the ohmic regimes, relatively close to the SCLC and TFLC regimes. This indicates that the LB device provides more controlled, reproducible, and stable charge transport across the analyzed voltage range compared to the disordered device.

## 4 Conclusions

The combined optical, vibronic, structural, and electrical analyses presented here provide a coherent picture of how MEH-PPV responds to aggregation, conformational disorder, and film-processing conditions. In solution, the Lambert–Beer law alone is insufficient to capture subtle structural variations. Instead, the behavior of the optical gap *E*_*optg*_ and the absorption band tail vs concentration reveals that aggregation effects first manifest in the band tail, probably when lateral interactions begin—likely dominated by side-chain interactions—before significant perturbations occur in the conjugated backbone. This observation is consistent with the fact that small variations in conjugation length (one or two monomer units) weakly affect *E*_*optg*_, whereas the Urbach tail is more sensitive to early stages of aggregation. Such aggregates can persist at the air–water interface and serve as heterogeneous nucleation centers, promoting further aggregate growth during lateral compression, as proposed by Nogueira et al.^6^ Consequently, the aggregation state in solution may directly influence the interfacial organization, domain formation, and subsequent film morphology.

The Langmuir–Blodgett (LB) deposition technique enables precise control of film thickness and reveals a strong interfacial influence in films up to approximately 10 layers, for which *E_u_* values are at least twice those observed in bulk-like films (Figure 7). In addition, the LB method induces partial orientational order in the polymer chains. Although *E*_*optg*_ remains essentially independent of polarization direction, absorption dichroism indicates that a larger number of conjugated backbones are aligned along the dipping direction. At the same time, *E_u_* shows no directional dependence, demonstrating that the distribution of shallow optical traps is equivalent parallel and perpendicular to the dipping direction, even when the population of aligned chromophores differs. Polarized emission measurements further confirm that LB deposition preferentially aligns chains with longer conjugation lengths along the dipping direction, with the mean conjugation length increasing by approximately two repeat units.

Analysis of vibronic parameters (the Huang–Rhys factor ***S***, mean conjugation length *n*, and vibronic energy separation *E*_00_ – *E*_01_) demonstrates that these quantities are sensitive probes of structural organization. LB films exhibit longer conjugation lengths and reduced ***S*** values along the dipping direction, consistent with enhanced chromophore alignment. Importantly, the vibrational mode coupled to the 01 emission depends on the deposition method: drop-cast films couple more strongly to the 1309 cm^–1^ mode (C=C stretching coupled to vinyl twisting), whereas LB films, particularly under 0°0° polarization, preferentially couple to lower-energy modes around 964 cm^–1^. These results show that molecular organization governs which vibrational modes become optically active.

Electrical characterization reveals that the two deposition methods produce films with markedly different trap landscapes. While both devices exhibit similar low-field exponents close to the ohmic regime, LB devices display smoother and more coherent transitions between ohmic, space-charge-limited current (SCLC), and trap-filled limit (TFLC) regimes. The earlier onset of the TFLC regime in disordered devices indicates a lower total trap density, whereas LB devices exhibit a higher trap density distributed over a narrower energy range, consistent with a more compact yet structurally constrained morphology. Crucially, the comparison between optical (Urbach) and electrical (TFLC) trap analyses demonstrates that these techniques probe fundamentally different trap populations: Urbach analysis is sensitive to shallow, optically active traps, while TFLC reflects deep, highly localized traps that are essentially invisible in UV–vis spectroscopy due to negligible wave function overlap.

In summary, the integration of absorption, emission, Raman spectroscopy, polarimetry, and electrical measurements provides a comprehensive understanding of the MEH-PPV morphology and disorder. The LB deposition method enhances partial orientational order, modulates vibronic coupling, and yields films with distinct trap distributions while preserving an almost polarization-direction-independent optical defect landscape. These findings highlight that morphological control via the deposition technique strongly influences vibrational coupling, excitonic structure, and charge-transport behavior, underscoring the necessity of combining optical and electrical probes to fully characterize disorder in conjugated polymer systems.

## Supplementary Material



## Notes

Figure S1: *π*–*A* isotherms; compression modulus $C_{s}^{-1}$ , film relaxation, and AFM image. Figure S2: Raman spectra. Table S1: Urbach Energy (*E_u_*) and optical band gap (*E*_*optg*_) in solution. Table S2: Gaussian parameters obtained by decomposition of the emission spectra at each different concentration, as well as Huang–Rhys, ***S,*** and *n* parameters. Table S3: Urbach Energy (*E_u_*) and optical band gap of LB and drop-casting films. Tables S4 and S5: Results and statistical analysis of fitting *I × F* curves (PDF)
