# Supplementary material for: Urbach Energy: Film Processing and Thickness Effects
Source: Langmuir. 2026 Apr 11;42(16):11100–12. doi: 10.1021/acs.langmuir.5c06770 (PMC13130963; doi:10.1021/acs.langmuir.5c06770)
Supplement: Supplementary file 1 [file la5c06770_si_001.pdf]

Supporting information

Urbach energy: Film processing and Thickness effects

Marleane Maria Felix de Azevedo[1], Endel Ezequiel Carvalho Costa[2], Guilherme Severino Mendes de Araújo[3], Kayla Manuelli Pimentel de Carvalho[1], Cleânio da Luz Lima[1], Ángel Alberto Hidalgo[1], Maria Leticia Vega[1].

Corresponding author: Hidalgo, Ángel Alberto

E-mail address: angel@ufpi.edu.br

Experimental Section

Raman spectra

To collect the Raman spectra, a SENTERRA II confocal Raman microscope from Bruker was used, the sample was excited using a 785-nm laser, and a diffraction grating with 400 lines/mm was used, which provides a spectral resolution with five increments of 10 s each. A 100x objective was used for the measurements, and the spectrum was normalized using vector normalization. All Raman measurements were taken at room temperature.

Langmuir and Langmuir-Blodgett films

Langmuir-Blodgett films were produced in a Langmuir trough KSV-2000 (Nima Technology). The polymer MEH-PPV was dissolved in chlorobenzene. The polymer was completely dissolved leaving the sample in a magnetic stirrer for 20 h at room temperature. Two different concentrations were tested to produce de Langmuir monolayers: 1.0 and 0.5 mg/mL. Figure S1 a) compares the isotherms with both concentrations and show the reproducibility of the isotherms with 0.5 mg/mL. We spread 70 µl for the 1.0 mg/mL and 120 µl for the 0.5 mg/mL of solution, onto the aqueous subface using a microsyringe. The spreading solution was deposited drop by drop and avoiding losing material to the subface.

Concentration of the spreading solution, i.e. 0.5 mg/mL, was selected on bases of the Langmuir $\pi - A$ isotherm. Results as good reproducibility, transfers ratio close to one and the absence of darker regions after spreading the

monolayer (indicative of possible multilayered regions) were the main results that oriented the spreading concentration and surface pressure for LB film transfer. Before initiating compression, we wait 10 min. to allow solvent evaporation. During the $\pi - A$ isotherms the barriers speed was set to 5 mm/min. Figure S1.c shows the monolayer stabilization. The barrier speed was set to 5 mm/min during the isotherm and film stabilization.

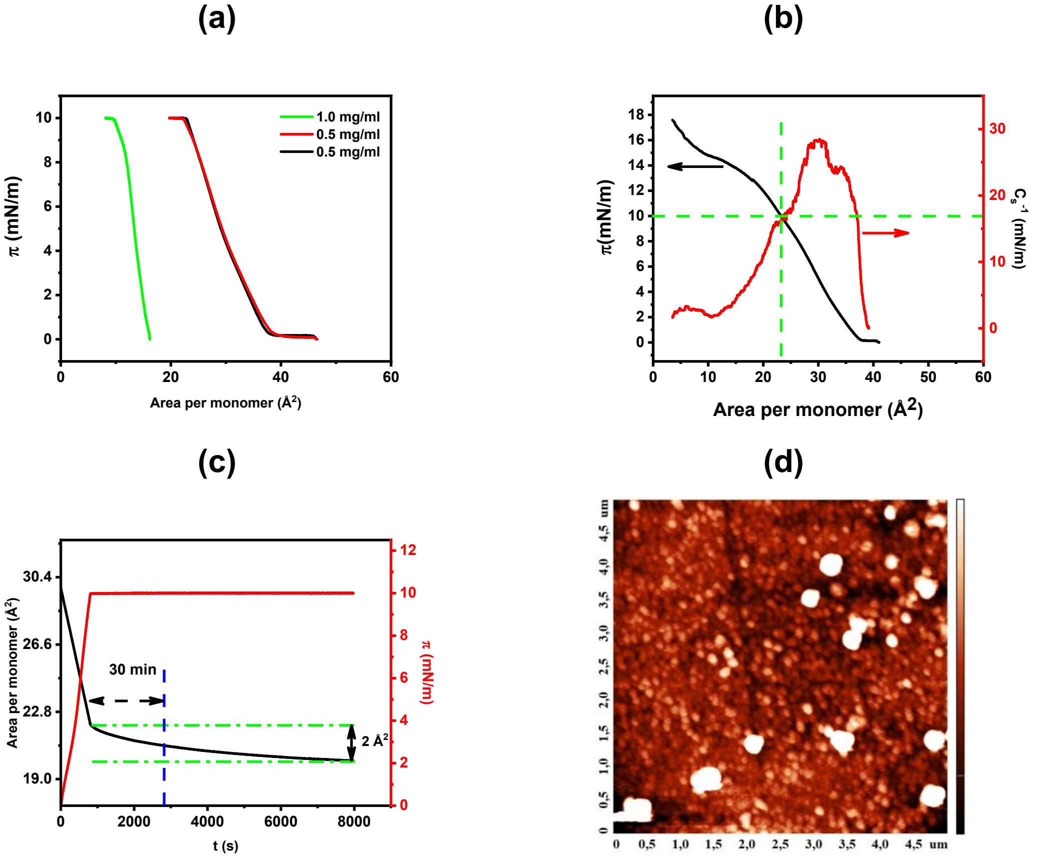

Figure S1: a) $\pi - A$ isotherms using 1 mg/mL and 0.5 mg/mL. The 0.5 mg/mL showed good reproducibility. **b)** Langmuir isotherm with the inverse of the compression modulus C_S^{-1}, the surface pressure π and C_S^{-1} for film transfer are signaled with dashed lines. **c)** film relaxation. Dot-dash lines show area change during relaxation at constant pressure and the dash vertical line indicate the instant when film transfer is initiated. **d)** show AFM image of an LB film with an area of 5 x 5 μm^2, showing homogeneous cover of the substrate.

1. MEH-PPV in solution

Table S1: Urbach Energy (E_u) and Optical band-gap (E_{optg}).

Conc. (mg/mL)	Urbach Energy (eV)	Optical band-gap (eV)
**0.33E-02**	0.026	2.25
**0.66E-02**	0.032	2.25
**0.98E-02**	0.037	2.25
**1.30E-02**	0.037	2.25
**1.61E-02**	0.038	2.25
**1.92E-02**	0.035	2.25
**2.23E-02**	0.035	2.25

Emission data as concentrations varies

Table S2: Gaussian parameters obtained by decomposition of the emission spectra at each different concentration as also Huang-Rys $\boldsymbol{S}$ and n parameters.

Conc. (mg/ml)	S	e00	e01	Area 00	E00	E01	n
		Energy position			Intensity		
**0.33E-02**	0.421	2.216	2.050	0.0253	0.159	0.067	8.78
**0.66E-02**	0.445	2.217	2.048	0.0251	0.155	0.069	8.65
**0.98E-02**	0.451	2.213	2.213	0.0252	0.153	0.069	8.62
**1.30E-02**	0.476	2.212	2.043	0.0250	0.151	0.072	8.51
**1.61E-02**	0.496	2.213	2.042	0.0247	0.147	0.073	8.41
**1.92E-02**	0.506	2.213	2.042	0.0247	0.146	0.074	8.37
**2.23E-02**	0.553	2.214	2.040	0.0241	0.139	0.077	8.16

2. Film characterization

Table S3: Urbach Energy (E_u) and Optical band-gap of LB and Drop-casting films.		
**LB film**	**Urbach Energy (eV)**	**Optical band-gap (eV)**
**F1C**	0.057	2.18
**F2C**	0.044	2.17
**F3C**	0.042	2.17
**F5C**	0.052	2.16
**F8C**	0.046	2.15
**F10C**	0.061	2.15
**F15C**	0.070	2.15
**F20C**	0.078	2.15
**Drop-casting Film**	**Urbach Energy(eV)**	**Optical band-gap (eV)**
**C_100 μL**	0.061	2.13
**C_125 μL**	0.075	2.13
**C_150 μL**	0.065	2.13
**C_175 μL**	0.067	2.13
**C_200 μL**	0.061	2.13

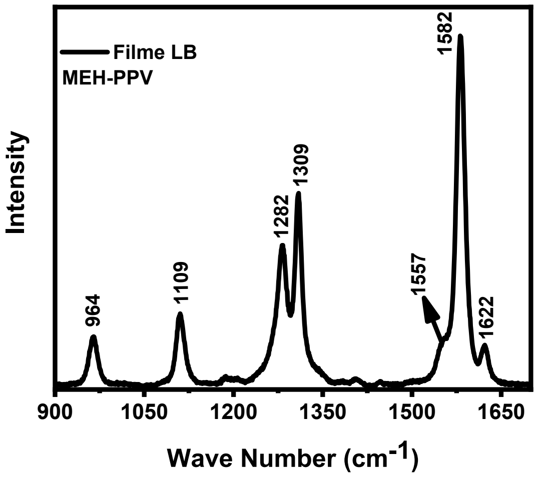

Figure S2: Raman spectra of LB film.

3. Linear fitting of the different regions of the JxV data

To select different regions for the linear fittings we used a combination of physical and statistical criteria. The analysis was performed on a log–log scale, in which different transport regimes in polymeric devices (Ohmic, SCLC and TFL) are theoretically expected to exhibit linear behavior. For each interval presented in Figure 8, linear fittings were performed and the exponent n, its associated uncertainty, and the coefficient of determination (R^2) and the adjusted coefficient of determination (R^2_{aj}) were systematically evaluated. Tables S4 and S5 show the data extracted from the fitting process.

Table S4: linear fitting results of the different regions in the partially ordered (LB) device

Region	Ohmic	TFLC
Slope n	1.3 ± 0.1	11.3 ± 0.3
R^2	0.961	0.995
R^2_{aj}	0.960	0.994

Table S5: linear fitting results of the different regions in the disordered (drop-casting) device

Region	Ohmic	TFLC	Trap-filled
Slope n	1.3 ± 0.2	12.0 ± 0.6	2.60 ± 0.03
R^2	0.867	0.983	0.987
R^2_{aj}	0.858	0.981	0.987
